# Retraction Note: Genome assembly of *M. spongiola* and comparative genomics of the genus *Morchella* provide initial insights into taxonomy and adaptive evolution

**DOI:** 10.1186/s12864-025-11667-x

**Published:** 2025-05-07

**Authors:** Qing Meng, Zhanling Xie, Hongyan Xu, Jing Guo, Qingqing Peng, Yanyan Li, Jiabao Yang, Deyu Dong, Taizhen Gao, Fan Zhang

**Affiliations:** 1 Qinghai University, 251 Ningda Road, Xining, 810016 Qinghai China; 2 Qinghai University of Technology, 15 Twenty-fourth Road, Xining, 810016 Qinghai China; 3 State-owned forest farms of Tianjun County, Delingha, 817299 Qinghai China; 4 Forestry and grassland station of Tianjun County, Delingha, 817299 Qinghai China

**Retraction Note: BMC Genomics (2024) 25:518**

10.1186/s12864-024-10418-8

The Editor has retracted this article because the authors were unable to provide evidence they had obtained the necessary permissions to use, in a comparative genomic analysis, 32 unpublished Morchella genomes sequenced by the Joint Genomic Institute. Figures 1, 3 and 4 using that data have now been removed. In addition, 30 pictures were used to illustrate Figs. 5 and 6 without proper credit. These figures have now been removed. The authors are invited to resubmit their article without the data used without permission and images that are based on such data. This submission will then be subjected to peer review. Qing Meng has stated on behalf of all authors they they agree to this retraction.

